# T‐antigen as a biomarker of progression‐free survival in patients with glioblastoma

**DOI:** 10.1002/acn3.52082

**Published:** 2024-05-09

**Authors:** Liao Guan, Wenwen Wang, Xuefei Ji, Hongwei Cheng, Weidong Du, Lei Ye

**Affiliations:** ^1^ Department of Neurosurgery the First Affiliated Hospital of Anhui Medical University Jixi Road 218 Hefei 230022 PR China; ^2^ First Clinical Medical College Anhui Medical University Meishan Road 81 Hefei 230032 PR China; ^3^ Department of Pathology Anhui Medical University Meishan Road 81 Hefei 230032 PR China

## Abstract

**Objective:**

Glioblastoma (GBM) is one of the most aggressive brain tumors and often leads to poor outcomes. Studies have indicated that glycan levels are significantly correlated with the pathogenesis and development of cancers. However, whether glycan levels can serve as diagnostic or prognostic biomarkers in GBM remains unclear.

**Methods:**

We obtained glycomic profiles in tissue and serum samples from 55 individuals with GBM using a well‐established lectin biochip platform probing with 11 specific lectins.

**Results:**

Our univariate analysis showed that 5 out of the 11 lectin‐probed glycans (LPGs) were significantly higher in GBM tissues than in peri‐tumoral tissues. After logistic regression analyses, only the Jacalin‐probed T‐antigen difference between the two groups remained significant (*p* = 0.037). Moreover, survival‐related analyses showed that the level of Jacalin‐probed T‐antigen was significantly associated with the progression‐free survival (*p* = 0.038) of patients. However, none of the LPG levels were correlated with the overall survival or the chemosensitivity to temozolomide therapy. The correlation coefficient analysis showed a moderate‐to‐strong correlation in the Jacalin‐probed T‐antigen levels between GBM tissues and serum samples, indicating its potential usefulness as a non‐invasive GBM progression biomarker.

**Interpretation:**

Glycomics analyses can be helpful in the prediction of GBM recurrences and may provide information useful for GBM glycan‐based target therapies or vaccine development.

## Introduction

Glioblastoma (GBM) is a frequent and invasive primary central nervous system (CNS) tumor in adults.^1^ According to the World Health Organization tumor classification, GBM is one of the highest graded gliomas featuring necrosis or microvascular hyperplasia.^2^ Multimodality treatments against GBM usually incorporate neurosurgery with subsequent radio‐ and/or chemo‐therapies. However, the overall GBM prognosis remains poor, with a median survival time of only 14.6 months.^3^ Therefore, identifying potential molecular targets that may predict or influence the outcomes of patients remains important.

Studies have identified molecules in tissue and serum samples that may help predict individual GBM prognoses. Swellam *et al*.^4^ found that circulating serum microRNAs could be a reliable biomarker for GBM diagnosis and prognosis. Other molecules, such as circulating tumor DNA,^5^ glial fibrillary acidic protein,^6^ extracellular vesicles,^7^ and exosomes,^8^ have been also proposed for GBM progression monitoring. The levels of these molecules are commonly influenced by both genetic and tumor microenvironment factors. However, the extensive factor heterogeneity and lack of standardized measurement platforms have led to controversial results. Therefore, novel biomarkers for prompt GBM assessments are urgently needed.

Unlike RNAs or proteins, which are synthesized following direct genetic templates, glycosylated molecules are formed via post‐translational modifications (PTMs) that are primarily influenced by microenvironmental or pathogenic factors in vivo.^9^ Importantly, glycans have been demonstrated to participate in various physiological and pathological processes, including cell adhesion, protein folding and trafficking, signal transduction, immune regulation, and molecular clearance.^10^ Associations between glycan levels and tumor progression have been reported: Hall *et al*.^11^ found that increased oligomannose glycan levels promote cell proliferation and invasion in neuroblastoma. In a tissue‐related glycoproteomic study, Trouillas *et al*.^12^ found a significant association between levels of polysialylated neural cell adhesion molecules and adverse survival outcomes in invasive pituitary adenomas, indicating that the molecules might be a useful prognostic biomarker. Moreover, functional glycan study results have suggested that component analysis of glycosylation may provide insights into the pathogenesis and disease progression of CNS tumors.^13^, ^14^ However, GBM histological and serologic glycomic profile studies are still needed.

For this study, we obtained the glycomic profiles from both tissue or serum samples of patients with GBM using a well‐established lectin microarray platform, which specifically recognizes carbohydrates that bind lectins in interactions similar to those in antigen–antibody complexes. We assessed potential correlations between glycan levels and temozolomide (TMZ) sensitivity or survivals. Moreover, we performed correlation coefficient analyses between glycans in tissue and serum samples to explore the possibility of using non‐invasive tests to measure biomarker levels. Our results may also help optimize cancer‐specific immunotherapies.

## Materials and Methods

### Patients and samples collection

We conducted a retrospective analysis of glycan levels in tissue and serum samples using a well‐established lectin biochip platform. We randomly recruited 55 patients with GBM who attended the First Affiliated Hospital of Anhui Medical University between January 2019 and October 2021. Tumor tissues and pair‐matched para‐tumor tissues were collected from the patients. The pair‐matched para‐tumor tissues were defined as brain tissue specimens resected at the border of GBM tissues. Pathologist confirmed the absence of tumoral cells from para‐tissues based on H & E staining morphology. In addition, peripheral serum samples were collected from all patients. A senior pathologist diagnosed all GBMs following the 2021 WHO Classification of Tumors of the Central Nervous System.^15^ After the tumor resections, all patients received radiotherapy, with at least a six‐course treatment of concomitant TMZ therapy. Table 1 lists the demographic and clinicopathological characteristics of the 55 patients. We excluded patients with low‐grade glioma or other intracranial disorders, such as cerebrovascular disease, traumatic brain injury, neurodegenerative diseases, or functional neuropathies from this study. Tissue samples were collected aseptically during craniotomies, and they were stored in liquid nitrogen. Serum samples were collected in the morning before the surgical operation and were subsequently stored in a − 80°C freezer. All patients declined to receive radio‐ or chemo‐therapies prior to their surgical treatment. The study protocol was conceived in accordance with the tenets of the Declaration of Helsinki and the Ethics Committee of the First Affiliated Hospital of Anhui Medical University approved it. All participants signed written voluntary informed consents for participation.

**Table 1:** Demographic and clinicopathological information of patients in this study.

Characteristics	All GBM patients (*N* = 55, %)
Age (years)	
Range	34–81
Mean ± SD	59.25 ± 10.34
Gender, *n* (%)	
Male	33 (60.0)
Female	22 (40.0)
Location, *n* (%)	
Frontal lobe	27 (49.1%)
Temporal lobe	16 (29.1)
Other	12 (21.8)
Tumor size (mm)	50.33 ± 13.80
IDH status, *n* (%)	
Non‐mutated	34 (61.8)
Mutated	10 (18.2)
Unknown	11 (20.0)
MGMT methylation, *n* (%)	
Methylated	12 (21.8)
Unmethylated	43 (78.2)
Mortality, *n* (%)	24 (43.6)
Lost to follow‐up, *n* (%)	10 (18.2)

### Extraction of tissue proteins

We carried all protein extraction steps following our published protocol. Briefly, all reagents and instruments exposed to the samples were pre‐cooled at 4°C. Phosphate‐buffered saline (PBS), RIPA lysis buffer, and phenylmethanesulfonyl fluoride (PMSF) were commercially obtained from Sigma‐Aldrich (MO, USA). Following standard extraction procedures, we rinsed tissues with cold PBS to remove bloodstains. Next, we lysed 30‐mg tissue samples in 300 μL of RIPA buffer with PMSF to a final concentration of 1 mM. Subsequently, we ground the samples using a 70 Hz pulverizer at 4°C for 90 s and then froze them at −80°C for 30 min. After thawing on ice for 30 min, we centrifuged the ground samples at 14,000 rpm for 20 min at 4°C. Finally, we individually collected supernatants and stored them at −20°C.

### Purification and preparation of Cy3‐labeled samples

Prior to the preparation of Cy3‐labeled samples, we isolated high‐abundance proteins to minimize the negative influence of non‐specific glycoproteins in the supernatants of tissues and sera. High‐abundance proteins, including albumin and IgG antibodies, were separated using a Spin Albumin and IgG Erasin kit (Sangon, Shanghai, China) according to standard protocols. Briefly, 10 μL of each serum or supernatant sample was fully mixed with 200 μL of pre‐cooled binding/washing solution. Each mixture was individually poured into the resin‐containing centrifugal columns and incubated at 4°C for 15 min on a horizontal shaker. We collected the filtrates from each column after centrifugation at 7500 rpm for 60 s at 4°C, and we transferred them back to column beds. These steps were repeated twice, before collecting the final filtrates. In addition, we added 200 μL of pre‐cooled binding/washing solution to each column and centrifuged them at 7500 rpm for 60 s at 4°C. Thus, we obtained samples containing approximately 400 μL of filtrate without high‐abundance proteins, and we used a BCA Protein Assay Kit (Spark Jade, Shandong, China) to quantify the purified sample proteins in each of them. After that, we labeled the samples with Cy3 following our previous method.^16^ A PD Mini Trap G‐25 column (GE, Massachusetts, USA) was used to remove free Cy3 and obtain the Cy3‐labeled samples.

### Immobilization of lectin on biochips

We carried out chemical surface modifications of the gold biochip surface according to an established protocol.^16^ Lectins, including *Lotus tetragonolobus* lectin (LTL, L‐1320), *Lens culinaris* agglutinin (LCA, L‐1040), *Narcissus pseudonarcissus* lectin (NPL, L‐1370), Jacalin (L‐1150), *Ricinus communis* agglutinin I (RCA‐I, L‐1080), Peanut agglutinin (PNA, L‐1070), *Vicia villosa* lectin (VVL, L‐1230), Wheat germ agglutinin (WGA, L‐1020), *Maackia amurensis* lectin I (MAL‐I, L‐1310), *Sambucus nigra* lectin (SNA, L‐1300), and concanavalin A (Con A L‐1000‐500), were commercially purchased from Vector Laboratories (CA, USA). We described these lectins, which specifically bind to different glycans, in our previous study.^17^ Each lectin was dissolved in 10 mM HEPES buffer (pH 8.5) with 0.001% BSA to a concentration of 1 mg/mL. The lectin solutions were individually spotted onto the biochip surface and incubated at 25°C for 2 h to form a lectin probe‐coated surface. The biochips were then rinsed in PBST buffer (pH 7.4) 3 times for 2 min and dried with a nitrogen stream.

### Glioma glycoprotein profiles of tissues and sera

We added Cy3‐labeled samples individually to lectin‐probed biochips and incubated them in a moist dark chamber at 25°C for 1 h. After washing the biochips with PBST buffer (pH 7.4) and drying them with a nitrogen stream, we scanned the biochips with a Luxscan™ 10 K‐A microscanner (Capitalbio, Beijing, China) at a 650 nm wavelength to detect Cy3. The fluorescence intensity obtained from the biochips was recorded.

### Statistical analysis

We conducted data analyses using SPSS statistical software (version 26.0, IBM). We expressed all continuous variables as means ± standard deviations (SDs) or interquartile ranges (IQRs). We applied a pair‐wise *t*‐test for the univariate analysis between tumoral and peri‐tumoral tissues. We further run the independent variables with statistical significance in the univariate analysis in a multivariate analysis. We generated receiver operator characteristic (ROC) curves to discriminate tumoral from peri‐tumoral tissues. The false positive rate (100% − specificity) was plotted against the true positive rate (sensitivity), and we calculated the area under the curve (AUC) as a reflection of diagnostic efficacy. We assessed survivals using Kaplan–Meier curves and compared them using the log‐rank test. Correlations were calculated using Pearson's correlation analysis. All *p* values in the study are two‐tailed, and we considered all *p* values <0.05 as statistically significant.

## Results

### Glycosylation profile of GBM tissues

**Figure 1:**
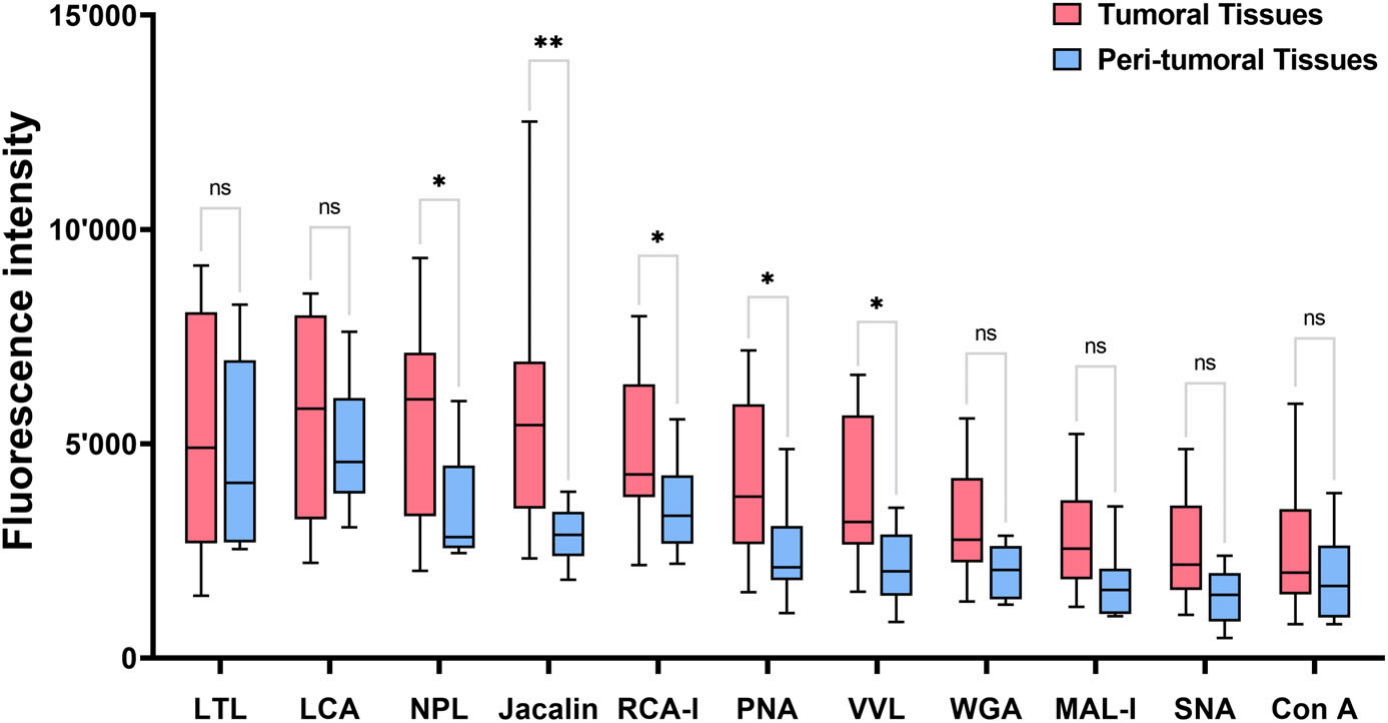
Univariate analyses for the expression differences of 11 lectin‐probing glycans between 55 pair‐wise tumoral tissues and peri‐tumoral controls in GBM patients. The levels of NPL (α‐Man), Jacalin (Galβ3GalNAc‐Ser/Thr), RCA‐I (Galβ4GlcNAc), PNA (Galβ3GalNAc), and VVL (GalNAc) were significantly upregulated in tumoral tissues in comparison with that in the peri‐tumoral tissues. Box in red: glioblastoma tissues; box in blue: peri‐tumoral tissues; NS, not significant; *p < 0.05; **p < 0.01.

We probed glycans in both tumoral and peri‐tumoral tissues of patients with GBM with 11 specific lectins on the biochip surface. Our univariate analysis results showed that 5 out of 11 lectin‐probed glycans (LPGs), including NPL (probing α‐Man) (*p* = 0.040), Jacalin (probing Galβ3GalNAc‐Ser/Thr) (*p* = 0.002), RCA‐I (probing Galβ4GlcNAc) (*p* = 0.046), PNA (probing Galβ3GalNAc) (*p* = 0.028), and VVL (probing GalNAc) (*p* = 0.030), were significantly upregulated in tumoral tissues when compared with their levels in peri‐tumoral tissues (Fig. 1). The differences of the remaining LPGs were not statistically significant between the two groups. Our multivariate analysis results revealed that the levels of the Jacalin‐probed glycan (*p* = 0.037) were significantly different between the two groups, indicating its potential as an independent biomarker (Fig. 1 and Table S1).

### Correlation analysis between LPG levels and overall survival (OS) or progression‐free survival (PFS) in GBM

First, we conducted a ROC analysis to obtain the cutoff levels for the five LPGs that were differentially expressed between tumor and para‐tumor tissues (Table 2). We classified the patients into high‐ and low‐expression groups accordingly. The patient numbers of high‐ and low‐levels of NPL‐probed glycan were 40 and 15; of Jacalin‐probed glycan was 38 and 17; of RCA‐I‐probed glycan were 44 and 11; of PNA‐probed glycan were 41 and 14; and of VVL‐probed glycan were 45 and 10, respectively.

**Table 2:** Receiver operator characteristic analysis for discriminating tumoral from peri‐tumoral tissues in patients with GBM.

Lectin‐probing glycan	Area under the curve	Cutoff value	Sensitivity (%)	Specificity (%)	Youden index	*p*‐value
NPL	0.789	4649.25	68.8	87.5	0.563	0.023
Jacalin	0.867	3950.75	68.8	100.0	0.688	0.004
RCA‐I	0.750	3673.00	81.3	87.5	0.688	0.050
PNA	0.781	2515.50	81.3	75.0	0.563	0.027
VVL	0.813	2413.50	81.3	87.5	0.688	0.014

**Figure 2:**
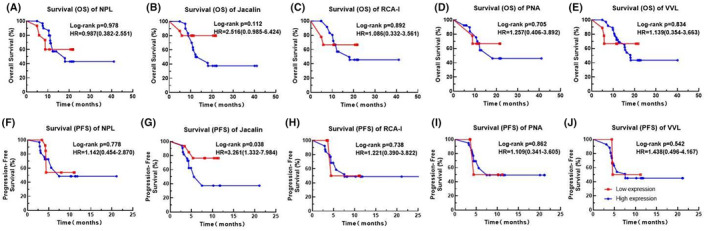
Overall survival (OS) (A–E) and progression‐free survival analyses (PFS) (F–J) in the patients with glioblastoma. Hazard risks and 95% confidence interval were estimated using a Cox proportional hazards model. Line in red: low expression; line in blue: high expression.

Next, we analyzed the potential correlation for these LPG levels with the OS or PFS of the patients (Fig. 2). None of these LPGs were correlated with the OS of our patients. However, the Jacalin‐probed glycan levels were significantly correlated with the PFS with a hazard risk of 3.261 (95% CI, 1.332–7.984; *p* = 0.038; Fig. 2G).

### Correlation analysis of LPG levels with clinicopathological parameters

We performed the correlation analyses of the clinicopathological parameters with the 5 LPG levels, which were differentially expressed between tumoral tissues and peri‐tumoral tissues. The results showed that Jacalin‐probed glycan was correlated with IDH mutation of GBM (*p* = 0.012); RCA‐I‐probed glycan was correlated with gender (*p* = 0.013); VVL‐probed glycan was correlated with tumor location (*p* = 0.046), respectively (Table 3).

**Table 3:** Correlation analysis of glycans in tissues and clinical features in GBM. High refers the high‐expression group and Low refers the low‐expression group.

	NPL			Jacalin			RCA‐I			PNA			VVL		
	High (*n* = 40)	Low (*n* = 15)	*p*‐value	High (*n* = 38)	Low (*n* = 17)	*p*‐value	High (*n* = 44)	Low (*n* = 11)	*p*‐value	High (*n* = 41)	Low (*n* = 14)	*p*‐value	High (*n* = 45)	Low (*n* = 10)	*p*‐value
Age (years‐old)	59.18 ± 9.23	59.20 ± 8.17	0.997	59.09 ± 9.28	59.40 ± 8.02	0.950	59.46 ± 8.92	58.00 ± 8.89	0.802	59.09 ± 9.28	56.00 ± 11.02	0.568	58.40 ± 9.48	57.40 ± 8.88	0.847
Gender (*N*)			0.537			0.557			0.013			0.206			0.498
Male	25	8		24	9		30	3		27	6		28	5	
Female	15	7		14	8		14	8		14	8		17	5	
Location (*N*)			0.122			0.418			0.191			0.126			0.046
Frontal lobe	22	5		20	7		21	6		17	10		25	2	
Temporal lobe	12	4		9	7		15	1		13	3		10	7	
Other	6	6		9	3		8	4		11	1		10	2	
Tumor size (mm)	54.25 ± 9.66	57.14 ± 16.57	0.663	56.39 ± 11.84	49.14 ± 11.72	0.267	54.72 ± 11.84	52.64 ± 14.61	0.793	53.56 ± 9.90	55.48 ± 15.08	0.755	55.94 ± 16.40	53.23 ± 8.28	0.659
IDH status (*N*)			0.120			0.012			0.682			0.459			0.210
Non‐mutated	28	6		26	8		28	6		27	7		26	8	
Mutated	6	4		3	7		7	3		6	4		8	2	
Unknown	6	5		9	2		9	2		8	3		10	0	
MGMT methylation (*N*)			0.477			0.158			0.230			0.259			0.199
Methylated	10	2		6	6		8	4		7	5		8	4	
Unmethylated	30	13		34	9		36	7		34	9		37	6	

### Assessment of the potential associations between LPG levels and TMZ efficacy against GBM

**Figure 3:**
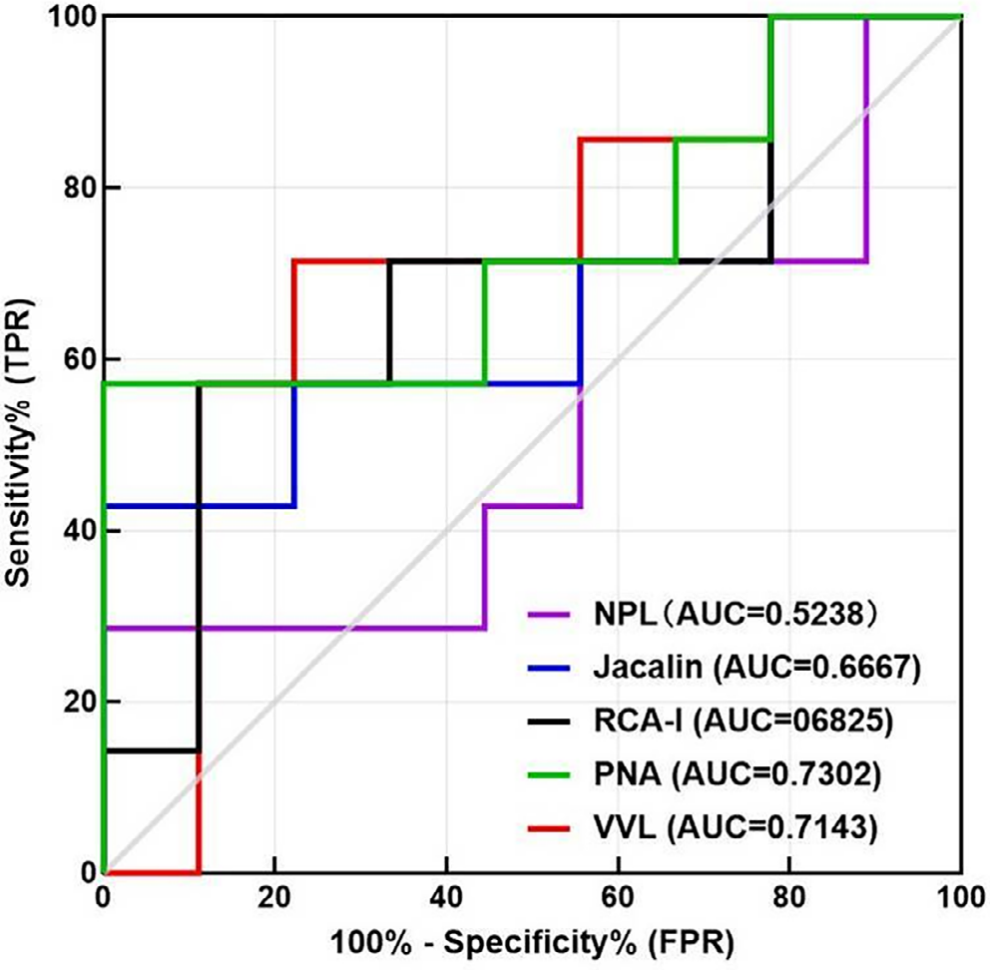
Receiver operator characteristic curves showing the predictive efficacies of lectin‐probed glycans in temozolomide chemotherapy in the patients with glioblastoma. FPR, false positive rate; TPR, true positive rate.

No standard definition of TMZ treatment efficacy exists due to the generally poor outcomes of patients receiving this treatment. Szczepanek *et al*.^18^ reported overall survivals of 16.0 months for patients receiving combined treatment of radiotherapy plus TMZ and 12.5 months for those receiving radiotherapy alone. The study suggested a mean survival time for patients receiving adjuvant TMZ treatment. Therefore, we defined the ineffective and effective TMZ treatments as those resulting in OSs shorter than 12.5 months and OSs longer than 12.5 months, respectively. We also assessed correlations between differentially expressed LPG levels in tissues and the TMZ chemotherapy efficacy using a ROC analysis (Fig. 3). However, the LPG levels were not statistically different between the effective and ineffective groups (the *p* values for NPL, Jacalin, RCA‐I, PNA, and VVL were 0.274, 0.266, 0.224, 0.125, and 0.153, respectively), indicating that LPGs may have a minor effect on TMZ chemosensitivity in patients with GBM.

### Correlation coefficient analysis of LPG expression levels between tissues and sera

**Figure 4:**
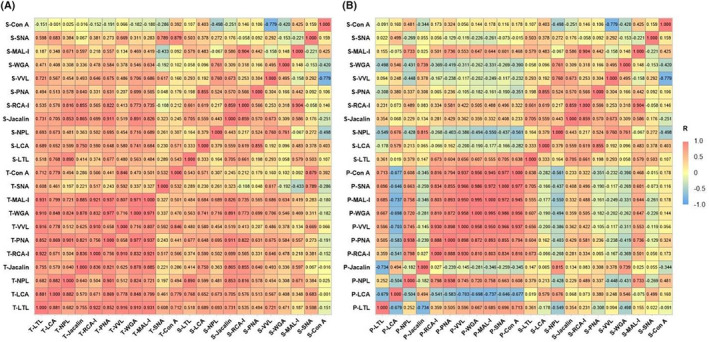
Correlation coefficient analyses of lectin‐probing glycans (LPGs) between tissues and sera in 55 patients with GBM. (A) the correlation analysis between the tumoral tissues and sera; (B) the correlation analysis between the peri‐tumoral tissues and sera. T‐lectin indicates the LPGs in tumoral tissues; S‐lectin indicates the LPGs in sera; P‐lectin indicates the LPGs in peri‐tumoral tissues.

We performed a Pearson's correlation analysis of LPG expression levels between tissues and pair‐wise serum samples (Fig. 4). Our results show that the LPG expression levels in sera were correlated more strongly with the corresponding levels in tumoral tissues than with those in the peri‐tumoral tissues. Moreover, we found moderate‐to‐strong positive correlations (correlation coefficient *r* > 0.75) of 2 LPGs between tumoral tissue and serum samples: Jacalin (*r* = 0.865) and SNA (*r* = 0.789). These results indicate that the levels of these LPGs in sera may reflect the corresponding levels in GBM tissues; consequently, serum levels may provide non‐invasive information for predicting GBM outcomes.

## Discussion

For this study, we obtained glycomic profiles of both tissue and peripheral serum samples of patients with GBM using a robust lectin biochip platform. Our logistic regression analysis results indicate that the level of Jacalin‐probed glycan was significantly higher in GBM tissues than that in peri‐tumoral tissues. In addition, Jacalin‐probed glycan levels were also correlated with the PFS of patients. However, none of these LPG levels were correlated with the clinical efficacy of TMZ chemotherapy. Finally, our correlation coefficient analysis results of LPG levels between tissue and serum samples revealed moderate‐to‐strong correlations for SNA‐ and Jacalin‐probed glycans, indicating that these glycans might be useful as non‐invasive biomarkers.

Protein glycosylation alterations are considered a universal feature of cancer cells, and the complexity of glycosylation increases the heterogeneity and functional diversity of tumors.^19^, ^20^ Glycosylation generates highly regulated cellular glycans that have effects on many biological processes.^21^ Studies have demonstrated that glycans and their derivatives accompany the development and progression of cancers and could potentially serve as biomarkers for diagnosis and prognosis. Huang *et al*. found that an N‐glycosylated B7 homolog 3 protein (B7H3) is significantly correlated with poor prognoses in patients with triple‐negative breast cancer (TNBC), and they demonstrated that the aberrant core fucosylation of B7H3 suppresses anti‐tumor immune responses in patients with TNBC^22^. Other studies have also shown that the altered O‐ or N‐glycosylation patterns of glycoproteins have important roles in tumor immunoregulation. Sialoglycans generate an immunosuppressive tumor microenvironment, contributing to blocking of T‐cell‐mediated killing of tumor cells and inducing tumor immune evasion.^23^, ^24^ Similarly, significant biological effects of glycans in numerous pathways of brain tumors have also been reported. GBM cells with increased α‐2,6‐sialylation levels exhibit strong progression and self‐renewal capacities.^25^ Moreover, Xu *et al*. found that N‐glycosylated N‐cadherin promotes tumor cell migration via inhibition of cadherin‐mediated cell–cell adhesion in gliomas.^26^

In our study, we found the levels of 5 LPGs (NPL, Jacalin, RCA‐I, PNA, and VVL) to be significantly higher in GBM tumoral tissues than in peri‐tumoral tissues. Studies have shown correlations between glycan levels and gliomas: Xu *et al*.^27^ reported that the levels of Galβ4GlcNAc, a glycan to which RCA‐I binds, are higher in astrocytomas than in normal brain tissues and are correlated with tumor malignancy. Moreover, Gc *et al*.^25^ found that the upregulation of β‐galactoside α2,6‐sialyltransferase 1 in GBM cells contributes to the increased growth and self‐renewal abilities of the cells. However, only the levels of our Jacalin‐probed glycan (Galβ3GalNAc‐Ser/Thr) remained significantly different between GBM and peri‐tumoral tissues after the logistic regression analysis. As early as 1984, Springer *et al*.^28^ reported that the levels of the Jacalin‐binding Galβ3GalNAc‐Ser/Thr, generally known as T‐antigen (Thomsen‐Friedenreich antigen), are correlated with the differentiation of carcinoma cells. In addition, the clustered T‐active structures on carcinoma cell surfaces have been involved in invasiveness, indicating that they could be useful during cancer diagnosis and immunotherapy.^28^ T‐antigen has been demonstrated to bind circulating galectin‐3 (Gal‐3), thereby inducing cancer cell polarization, so that the cell adhesion molecules exposed enhance tumor cell homotypic aggregation and prevent anoikis.^29^, ^30^ Moreover, Glinsky *et al*.^31^ reported that T‐antigens attach breast and prostate cancer cells onto endothelia by specifically interacting with endothelium‐expressed Gal‐3. Therefore, the interaction between the T‐antigen and Gal‐3 seems to have a pivotal role in the initial adhesion and proliferation of cancer cells. Macaluso *et al*.^32^ reported that T‐antigens induce or maintain a chronic endoplasmic reticulum (ER) stress state serving as potential mediators of non‐canonical ER‐dependent death pathways in mouse medulloblastomas. However, direct evidence for a mechanism linking T‐antigens and gliomas is missing. Interestingly, studies demonstrated that JC virus, a human polyomavirus, and its derivate T‐antigens are present in gliomas in both clinical tissues and glioblastoma xenografts of animals.^33^, ^34^, ^35^ In our study, we could not distinguish whether the Jacalin‐probed T‐antigen was a product of post‐translational modification of endogenic proteins or a derivate of the JC virus. The origin of T‐antigens should be clarified in another study.

We found a moderate‐to‐strong Jacalin‐probed T‐antigen correlation between the levels in GBM tissues and those in serum samples. This result suggests that the levels of Jacalin‐probed T‐antigen in serum may be similar enough to those in GBM tissues to offer a non‐invasive route for measuring the biomarker levels for PFS prediction in patients.

In addition to their clinical predictive role, glycomic profiles of tumoral tissues are also potentially crucial for development of glycan‐based medications or anti‐tumor vaccines. Aberrant glycosylation patterns generate tumor‐specific antigens that can be exploited for glycan‐based immunotherapies.^36^ Monoclonal antibodies (mAb) targeting carbohydrate chains have attracted attention as a modern medical technology for clinical diagnoses and treatments of cancer (possibly even after the failure of standard therapies). Disialoganglioside (or ganglioside D2, GD2), a glycoconjugate on the surface of tumor cells, has been confirmed to be expressed at high levels in neuroblastoma. Based on this finding, two anti‐GD2 mAbs have been developed, and the US Food and Drug Administration has approved them as cancer therapeutics.^37^ Other anti‐glycan mAbs have been under phase I or II clinical trials, such as hu14.18K322A for neuroblastoma targeting ganglioside GD2,^38^ DS‐3939 ADC for ovarian cancer targeting Tn,^39^ and OBI‐999 for advanced solid tumors targeting Globo H^40^. We measured the levels of only a few glycans, but our results provide a novel venue for immunotherapeutic strategies against GBM.

We are aware of the limitations of our study: First, we focused on a small cohort of patients with GBM, and the sample size should be augmented to confirm our conclusions. Second, we failed to explore the underlying causality in the associations between the glycans and GBM biological behaviors. An extensive study is needed to investigate the specific regulatory mechanisms of glycans in GBM.

## Conclusion

We uncovered the glycomic profiles of tissues and sera of patients with GBM using a well‐established lectin biochip. Our results suggest that serum Jacalin‐probed T‐antigen levels, which were positively correlated with those in GBM tissues, may be used as a non‐invasive biomarker of PFS, predicting GBM recurrences.

## Funding Information

This study was funded by the National Natural Science Foundation of China (grant No. 81901238), Scientific Foundation of Anhui Medical University (2022xjk144), Natural Science Foundation of Anhui Province (2208085MH224), and Co‐construction Project of Anhui Medical University and Affiliated Hospital (grant No. 2021lcxk017).

## Conflict of Interest

The authors declare no competing interests.

## Author Contributions

Liao Guan, Wenwen Wang, and Xuefei Ji conceived the research, drafted the manuscript, and performed the statistical analysis. Xuefei Ji and Wenwen Wang performed the experiment and made the interpretation of the results. Hongwei Cheng administered the project. Lei Ye revised the manuscript.

## Supporting information


Table S1.

## Data Availability

The original data presented in the study are included in the article file, and other datasets generated and/or analyzed during the current study are available from the corresponding author on a reasonable request.
