# Supplementary material for: T‐antigen as a biomarker of progression‐free survival in patients with glioblastoma
Source: Ann Clin Transl Neurol. 2024 May 9;11(7):1765–74. doi: 10.1002/acn3.52082 (PMC11251471; doi:10.1002/acn3.52082)
Supplement: Supplementary file 1 — Table S1. [file ACN3-11-1765-s001.docx]

**Supplementary Table 1.** Univariate analysis and multivariate logistic regression analysis of lectin probing glycans in GBM. B: partial regression coefficient;

|  | **Univariate analysis** | **Logistic regression model** | | | |
| --- | --- | --- | --- | --- | --- |
|  | ***P* value** | **B** | **OR** | **95% CI** | ***P* value** |
| **NPL** | 0.040 | 0.009 | 1.009 | 0.819-1.241 | 0.671 |
| **Jacalin** | 0.002 | 0.071 | 1.073 | 1.013-1.137 | 0.037 |
| **RCA-I** | 0.046 | 0.058 | 1.060 | 0.902-1.244 | 0.139 |
| **PNA** | 0.028 | - 0.011 | 0.989 | 0.577-1.658 | 0.461 |
| **VVL** | 0.030 | 0.051 | 1.052 | 0.897-1.233 | 0.292 |
